# Hierarchical and Nonlinear Dynamics in Prefrontal Cortex Regulate the Precision of Perceptual Beliefs

**DOI:** 10.3389/fncir.2019.00027

**Published:** 2019-04-24

**Authors:** Leonardo L. Gollo, Muhsin Karim, Justin A. Harris, John W. Morley, Michael Breakspear

**Affiliations:** ^1^QIMR Berghofer Medical Research Institute, Brisbane, QLD, Australia; ^2^Centre of Excellence for Integrative Brain Function, QIMR Berghofer Medical Research Institute, Brisbane, QLD, Australia; ^3^School of Psychiatry, Faculty of Medicine, University of New South Wales, Sydney, NSW, Australia; ^4^The Black Dog Institute, Sydney, NSW, Australia; ^5^School of Psychology, The University of Sydney, Sydney, NSW, Australia; ^6^School of Medicine, Western Sydney University, Sydney, NSW, Australia; ^7^Metro North Mental Health Service, Brisbane, QLD, Australia; ^8^Hunter Medical Research Institute, University of Newcastle, New Lambton Heights, NSW, Australia

**Keywords:** decision making, dynamic causal modeling, fMRI, prefrontal cortex, vibrotactile

## Abstract

Actions are shaped not only by the content of our percepts but also by our confidence in them. To study the cortical representation of perceptual precision in decision making, we acquired functional imaging data whilst participants performed two vibrotactile forced-choice discrimination tasks: a fast-slow judgment, and a same-different judgment. The first task requires a comparison of the perceived vibrotactile frequencies to decide which one is faster. However, the second task requires that the estimated difference between those frequencies is weighed against the precision of each percept—if both stimuli are very precisely perceived, then any slight difference is more likely to be identified than if the percepts are uncertain. We additionally presented either pure sinusoidal or temporally degraded “noisy” stimuli, whose frequency/period differed slightly from cycle to cycle. In this way, we were able to manipulate the perceptual precision. We report a constellation of cortical regions in the rostral prefrontal cortex (PFC), dorsolateral PFC (DLPFC) and superior frontal gyrus (SFG) associated with the perception of stimulus difference, the presence of stimulus noise and the interaction between these factors. Dynamic causal modeling (DCM) of these data suggested a nonlinear, hierarchical model, whereby activity in the rostral PFC (evoked by the presence of stimulus noise) mutually interacts with activity in the DLPFC (evoked by stimulus differences). This model of effective connectivity outperformed competing models with serial and parallel interactions, hence providing a unique insight into the hierarchical architecture underlying the representation and appraisal of perceptual belief and precision in the PFC.

## Introduction

Percepts underpin all our interactions with the world. Perceptual precision, the confidence with which we hold those percepts, informs this interaction, such as when a decision is biased toward a precisely represented percept (Ernst and Banks, 2002). Although high perceptual precision may be advantageous in some contexts, such as when driving a car, there exist other situations where a degree of imprecision is crucial: if percepts were held with infinite precision then it would be impossible to recognize any object encountered for a second time. For example, the texture of a surface would feel unique and surprising on every touch. Whereas the neurobiology of perception has been a long-studied subject, research into the basis of perceptual precision and its impact on decision making has been more recent (Knill and Pouget, 2004; Moran et al., 2013; Pouget et al., 2013; Navajas et al., 2017).

**Figure 1:**
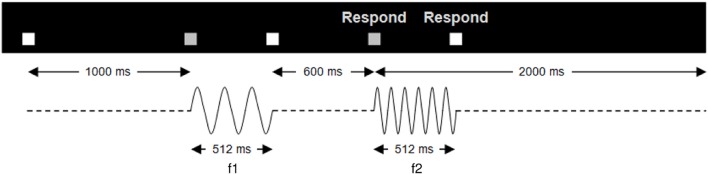
Trial structure. Temporal structure of a single trial of the vibrotactile discrimination task. A pair of stimuli (f1, f2), each 512 ms in duration, separated by an ISI of 600 ms, was presented to the participant’s right index finger. The start of the trial was indicated by a white box, which turned gray when the vibrations were presented. Upon the onset of the second vibration, a respond screen appeared indicating that the participant could make a button press. Participants had 2 s in which to respond after the second vibration onset. Trials were presented in four sessions; two sessions of faster-slower and two same-different.

**Figure 2:**
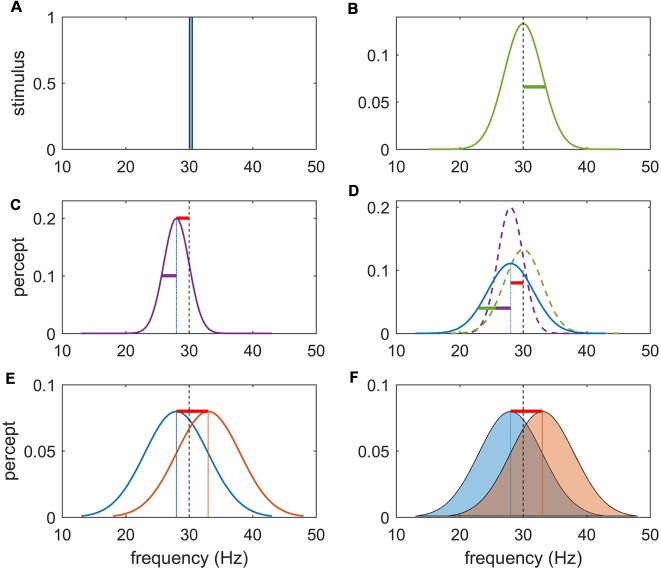
Schema for task rationale. **(A)** Frequency content of a noise-free stimulus of 30 Hz. **(B)** Noise imbued vibrotactile stimulus with center frequency of 30 Hz and variance of stimulus noise represented by the green bar. Precision refers to the inverse of the variance of the percept. **(C)** Perceptual encoding of a noise-free stimulus can be represented by a unimodal distribution centered at the likely value of the inferred stimuli. Note that due to an inevitable perceptual error (bias) this inferred stimulus is shifted to the left (or right) of the true stimulus frequency (red bar) and has perceptual noise (purple bar). **(D)** Perceptual representation of a noisy stimulus can be conceptualized as the sum of the stimulus (external) noise (green) and the perceptual (internal) noise (purple). It may have a perceptual bias (red bar) and perceptual noise (purple bar) in addition to stimulus noise (green bar). In separate sessions, participants were either instructed to answer the question “Is the 2nd vibration faster?” or “Are the vibrations different?” as a yes/no response. **(E)** The first task can be solved by subtracting the values of the inferred stimulus and responding on the sign of the answer. **(F)** The second task requires that the inferred magnitude of this difference be weighted by the precision (inverse variance) of each percept. Due to the perceptual error, there will exist a difference in the inferred frequency difference even if f1 = f2.

The neural basis of perceptual decision-making has been extensively studied using two-alternative forced-choice tasks in the somatosensory (Romo and Salinas, 2003) and visual domain (Britten et al., 1992). These prototypical experiments consist in presenting two sequential stimuli that are followed by a forced response between two choices involving a comparison between the properties of these two stimuli (see Figure 1). In the somatosensory modality, a wealth of neurophysiological research using vibrotactile stimuli has established the crucial role of the prefrontal cortex (PFC) during the performance of such tasks (Gold and Shadlen, 2007; Hegner et al., 2007; Heekeren et al., 2008; Wang, 2012). While the primary somatosensory cortex is clearly involved in stimulus representation (Hernández et al., 2000; Harris et al., 2002; Sörös et al., 2007), the PFC holds the representation of the first stimulus in working memory for subsequent comparison against representation of the second stimulus (Preuschhof et al., 2006; Wang, 2008), as well as the final decision process (Miller et al., 2003; Pleger et al., 2006; Heekeren et al., 2008; Wang, 2008; Barak et al., 2010). With very few exceptions (Engel and Wang, 2011), decisions in these forced-choice experiments are only dependent on magnitude comparisons of the perceived frequencies. A sensory percept can be viewed probabilistically (as a probability distribution) and to first order can hence be decomposed into its magnitude (here, the perceived frequency) and its precision (the inverse of the variance of the probability distribution; see Figure 2). Whilst perceptual precision—classically captured by the signal-to-noise ratio—impacts upon the performance accuracy of a faster-slower comparison, the decision itself does not explicitly require representing and acting on the precision of those perceptions. This is because the final decision only rests upon deciding whether the second stimulus is faster or slower than the first and does not depend upon the subjective confidence in that judgment. That is, a faster-slower decision can be made by a simple subtraction and does not crucially depend upon the precision of either percept.

The anterior cingulate and ventromedial PFC appear to play critical roles in assessing the value of current information in an environment of uncertain outcome and reward (Daw et al., 2005; Kennerley et al., 2006; Behrens et al., 2007). These regions also represent changes in this value (that is, when the link between stimulus, outcome and reward is volatile; Rushworth and Behrens, 2008). Whilst the value of the percept to an external reward is uncertain in these studies (Fiorillo et al., 2003; Yu and Dayan, 2005; Hsu et al., 2005; Huettel et al., 2006; Behrens et al., 2007; Tobler et al., 2007), the percept itself is not ambiguous. Hence, it is not clear from these studies whether these regions are also involved in representing the intrinsic precision of the percept itself, or whether other regions are recruited when the stimulus is noisy but the task contingencies are fixed (Kayser et al., 2010; Bach and Dolan, 2012).

Here, we sought to disentangle the representation of stimulus properties from the precision of those representations in the PFC. Functional neuroimaging data were acquired whilst paired vibrotactile flutter stimuli (10–50 Hz) were sequentially applied to the index finger. In separate tasks, participants were requested to decide if the second stimulus was *faster* than the first, or if the second stimulus was *different* from the first. As rehearsed above, the “faster-slower” task can be performed by simply encoding and subtracting an estimate of each stimulus frequency—that is, decisions only explicitly depend on comparing the likely value of each of the flutter frequencies. In the “same-different” task, the magnitude of this subtraction must be weighed against the precision of the perceptual beliefs, such that a difference that is perceived as small may be inferred as significant if each percept is held precisely (and conversely for imprecise representations). The precision of a percept is the composite of the roughness of the stimulus and the perceptual imprecision due to stochastic effects in perceptual systems: to manipulate stimulus precision, noise was introduced to the vibrotactile oscillatory frequency as an additional experimental factor (Harris, 2006; Harris et al., 2006; Karim et al., 2012). Note that we refer to precision in the statistical sense of the inverse of the noise variance (Figure 2).

The PFC is known to be underpinned by extensive intrinsic anatomical connections, forming local circuits that adapt to contextual demands at hand (Fuster, 2001; Miller and Cohen, 2001; Botvinick, 2008). The hierarchical nature of these circuits during the representation of perceptual precision is poorly understood (Nee and D’Esposito, 2016). We first identify a constellation of regions in the left PFC that respond to these stimulus and task manipulations. We then study the prefrontal networks that underpin our data using dynamic causal modeling (DCM). DCM is a model-based technique to infer network dynamics (Friston et al., 2003) that has found explanatory utility in cognitive neuroscience, including language (Leff et al., 2008; Noppeney et al., 2008), motor processes (Grefkes et al., 2008), vision (Mechelli et al., 2003; Fairhall and Ishai, 2007) and memory (Smith et al., 2006). DCM has been employed to study perceptual decision-making tasks (Summerfield et al., 2006; Stephan et al., 2007; Summerfield and Koechlin, 2008) including vibrotactile discrimination tasks, focussing on the exchange of information from primary to secondary somatosensory cortex (Kalberlah et al., 2013). Here, we use DCM to disambiguate between candidate serial, parallel or hierarchical engagement of the PFC in the representation and manipulation of perceptual precision.

## Materials and Methods

### Overview

Sixteen healthy young adults participated in our experiment. To avoid ceiling or floor effects and reduce inter-subject variability in performance, participants first performed an adaptive staircase procedure. Behavioral and functional imaging data were then acquired while they performed the main vibrotactile experiment. Analyses of these data then informed the employment of DCM. Each of these steps is described below. Full details are provided in the Supplementary Material.

### Participants

Sixteen healthy volunteers (10 men; mean age, 28.4 years; standard deviation, 9.3; age range, 20–61 years) participated in the study. Participants gave written informed consent and the study was approved by the University of New South Wales Human Research Ethics Committee. Participants were paid for their participation in the study. All participants were right-handed. Participants disavowed history of a psychiatric disorder, neurological disorder, or drug or alcohol dependence. Participants gave written informed consent according to local institutional human ethics committee approval.

### Stimuli and Task

Using an MR-compatible stimulator, mechanical vibrotactile stimuli were delivered to the right index finger (see Supplementary Material, SM1.1). Trials consisted of a series of paired stimuli, each 512 ms in duration, separated by an ISI of 600 ms (Figure 1 and Supplementary Material, SM1.2).

### Titration Procedure

To limit individual variability in performance and avoid ceiling effects in accuracy, we used a titration procedure that matched average task performance *via* an adaptive staircase procedure as described previously (Karim et al., 2012). The participants responded to the question: “Is the 2nd vibration faster?” For each trial, one of the vibrations was the base 34 Hz, and the other a comparison vibration, which varied based on the participant’s current performance according to an adaptive staircase procedure. The presentation order of the base and comparison was pseudorandomly varied from trial to trial.

Two intermixed staircases (easy and hard) selected at random were used to limit the participant from experiencing a learning effect from consecutive easy or consecutive hard trials. The difference in frequency between vibration pairs was initially set to 5 Hz, then progressively decreased or increased by 10% of the current frequency difference. For both staircases, a step-up occurred for each incorrect response. For the easy staircase, a step-down occurred after six non-consecutive correct responses. That is, even amongst trials of incorrect responses, a tally was kept for each correct response made. Once the tally reached six, a step-down occurred and the tally was reset to zero. Likewise, for the hard staircase, a step-down occurred after two non-consecutive correct responses. We sought to have performance converge at ~90% and ~65% proportion correct, respectively (Zwislocki and Relkin, 2001). A medium value of difficulty (target accuracy of 75%) was determined by calculating the geometric mean between the easy and hard frequency differences (Karim et al., 2012).

### Behavioral Task

Following titration, participants completed a parametric vibrotactile discrimination task with factors of context, noise and difficulty. “Context” denotes the task instructions—the faster/slower or the same/different comparison; “noise” refers to the presence or absence of random fluctuations in the stimuli. “Difficulty” refers to the (titrated) difference between the stimulus frequencies.

To create the noise factor, the temporal structure of the two vibrations was degraded by adding independent Gaussian-distributed values (mean = 0) to the wavelength of each cycle of the sine wave (Harris et al., 2006). We added 8% noise so that the standard deviation of the cycle length within the vibration equalled 0.08 of the base cycle length. For example, a 40 Hz vibration was comprised of cycles with mean length 25 ms and standard deviation of 2 ms. We hence refer to all trials as “regular” (noise-free) or “noisy.”

The contextual (task) factor was created by asking participants to perform either a fast-slow or a same-different comparison. In the fast-slow task, participants were instructed to answer the question “Is the 2nd vibration faster?” as a yes/no response. They were informed that there was always a faster vibration (i.e., no identical trials). In the same-different task, participants were instructed to answer the question “Are the vibrations different?” as a yes/no response. They were (correctly) informed that half of the presented vibration pairs were the same and half were different. Different trials in the second (same/different) context were identical to the corresponding trials in the first (faster/slower) context. For same-noisy trials in the second context, exactly the same stimulus was presented—that is, both the center frequency and the exact same pseudorandom sequence of jittered wavelengths. The rationale for our task design is illustrated in Figure 2.

For feasibility issues, not all cells in the full factorial design were performed. For example, in pilot testing, the accuracy of hard-noisy trials was at chance (50%) and was thus not used. We refer to the task as a “partial” factorial design in this sense. We do not report on the effect of task difficulty in this article and hence collapse all available trials (of equivalent difficulty) across this factor (for further details, see Supplementary Material, SM1.3 and Supplementary Table S1).

### MRI Acquisition and Analysis

Functional imaging data were acquired using a Philips (Achieva X) 3.0-Tesla scanner (for acquisition details see Supplementary Material, SM1.4). Stimuli were delivered *via* the vibrotactile device to the right index finger. Participants made button press responses *via* their left index and middle fingers. Inter-trial intervals were pseudorandomly jittered between 6 and 12 s to decorrelate the evoked hemeodynamic responses between trials. The task was conducted over four separate sessions separated by a short break. Each block consisted of exclusively same-different or faster-slower trials. Pre-processing of dynamic images included realignment, normalization, re-sampling and spatial smoothing using SPM8. Statistical analysis of the time series of images was conducted using the General Linear Model (GLM; Friston et al., 1994a) with regressors modeling each of the factor components. To focus on the decision-making process, we used a boxcar of width 200 ms immediately prior to the button press response. The results reported here are robust to changes in the width of the regressor. These were convolved with the canonical hemeodynamic response function.

Group-level, random-effects analyses used a flexible factorial analysis of variance (ANOVA) including a subject factor and non-sphericity correction for repeated measures (i.e., inhomogeneity of variance among conditions was estimated with ReML). In the second (same-different) task there also exists an additional stimulus factor, namely “Different” vs. “Same” trials: we hence also investigate this factor within this context. Statistical inference was performed at the cluster-level using family-wise error (FWE) correction, *p* < 0.05 (Friston et al., 1994b, 1996). Unless otherwise stated, we employed a height threshold of *p* < 0.00005 and a spatial extent of 20 voxels. All *p*-values reported in the Results are FWE-corrected. Cluster locations were identified using the SPM Anatomy toolbox (Eickhoff et al., 2005).

### Dynamic Causal Modeling

#### Model Specification

DCM is a computational approach that allows construction and comparison of dynamic network models of functional imaging data (Friston et al., 2003). DCM uses the time series from imaging data and combines a model of the hidden neuronal dynamics with a forward model that translates neural states into predicted measurements (Stephan et al., 2008). Specifying dynamic causal models requires two steps: first, regions (network “nodes”) that express the specific effects of interest (noise, context, same-different) are identified using the preceding GLM. These are described in the “Results” section, following analysis of the main and the interaction effects in our experiment. The time series data from each node are then extracted. We used a sphere of 6 mm radius centered at the voxel showing the group-wise maximum contrast (see Supplementary Material, SM1.5.1).

The second step in DCM specification involves the construction of a space of models that embody various hypotheses about the manner in which these nodes interact—that is, the (effective) connectivity, or network “edges,” between the nodes. Restricting the space of models to a relatively small family that test specific hypotheses is an important way to constrain the number (and utility) of models to be tested (Stephan et al., 2010). Since the present objective was to use DCM to study the network models of perceptual precision (hence, not focussing on basic vibrotactile processing *per se*), we restricted our analyses to a small number of models that shared a common sensory input base and added candidate integrative mechanisms on top of this base. The input base was the sensory area showing the main effect of stimuli, hence identified using an F-contrast across all trials. We introduced eight separate models (four bilinear and three nonlinear) on top of the common base that modeled serial or parallel integrative mechanisms. Serial, parallel or hierarchical architectures play varying roles in a diversity of cognitive and even machine learning systems (Mesulam, 1998; Friston, 2005; Petersen and Sporns, 2015): their disambiguation here, using DCM, can hence contribute to this broader literature, whilst also establishing the relative primacy of perceptual value vs. precision underlying decision-making in the presence of stimulus noise. These DCM’s each embody one of these arrangements, differing within-class according to the presence or absence of symmetrical relationships (see Figure 5, and results for a representation of the specified models). Nonlinear models specify hierarchical relationships between the network nodes—that is, where the neuronal activity in one region gates the flow of activity between other regions (Stephan et al., 2010); bilinear models mirror their more complex nonlinear counterparts, except they lack hierarchical relationships between regions: this gating (interaction) function is instead fulfilled by non-specific modulatory inputs.

**Figure 3:**
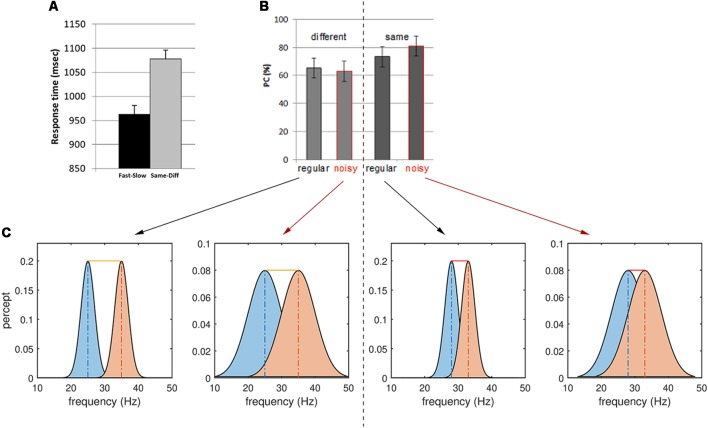
Behavioral results for the same-different context and interpretations. **(A)** Reaction time for Fast-Slow vs. Same-Different comparisons. Note the longer reaction times for the latter task. **(B)** Proportion of correct (PC) responses (or accuracy) of regular and noisy response for different and same trials in the Same-Different task. **(C)** Stimulus noise increases the variance of the perceptual representation of the two frequencies f1 and f2, increasing the overlap between them. A larger overlap between perceptual representations decreases the sensitivity of responses to Different trials (left). The yellow bar depicts the difference between the mean of the two percepts—here the sum of the true stimulus differences and the perceptual error. Conversely, noise increases the accuracy of responses to Same trials (right): some slight difference in perception occurs even for identical, periodic stimuli (red bars, sum of perceptual errors). However, stimulus noise degrades the precision of each percept, hence increasing their overlap and masking these small (false) perceptual differences.

**Figure 4:**
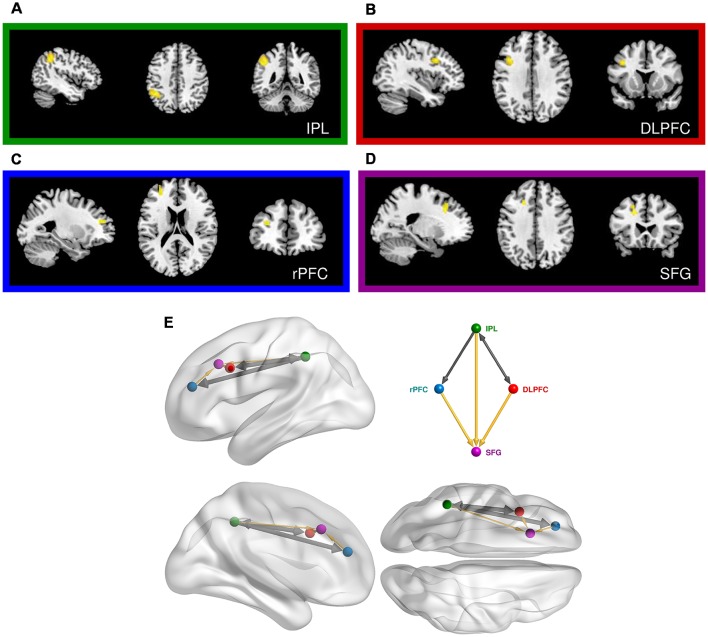
Prefrontal cortical regions engaged in the same-different vibrotactile trials. **(A)** Main effect of the “Different > Same” contrast in the left inferior parietal lobe (IPL). **(B)** Main effect of the “Different > Same” contrast in the left dorsolateral prefrontal cortex (DLPFC). **(C)** Main effect of “Noise-free > Noisy” contrast in the left rostral PFC (rPFC). **(D)** Interaction of noise and difference in the left superior frontal gyrus (SFG). **(E)** Relative anatomical location of the corresponding nodes employed in the dynamic causal modeling (DCM), as labeled and colored in the inset. Thick arrows show effective connectivity common to all DCM models. Thin yellow links show connections used in some but not all models.

**Figure 5:**
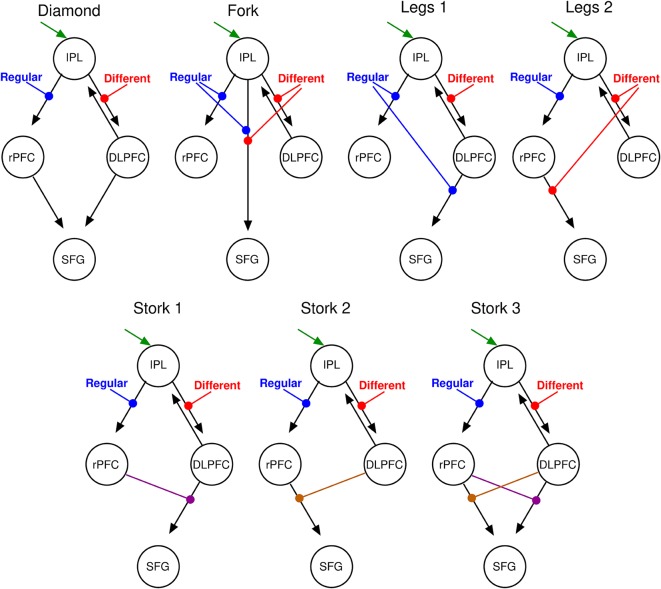
DCM parsimonious and non-redundant model space. Stimulus inputs arrive *via* the IPL (green arrow) and propagate, *via* intrinsic connections (black arrows) to the rPFC and the dorsolateral PFC (DLPFC). Each of these intrinsic connections is perturbed by experimental inputs: different (red) and Regular (blue) stimulus trials that account for the corresponding effects in the SPM contrasts. From left to right, top motifs are linear serial (Diamond), parallel (Fork), hierarchical with the regular modulation at the higher level (Legs 1), and a hierarchical with the different modulation at the higher level (Legs 2). Bottom motifs are hierarchical and nonlinear. From left to right, regular modulation is at the top of the hierarchy (Stork 1), different modulation is at the top of the hierarchy (Stork 2), and the double non-linear model in which both modulations occupy top and low hierarchy positions at the different interactions (Stork 3). Please refer to the Supplementary Material for further discussion on the model space.

#### Model Selection and Parameter Estimation

Following model specification, DCM employs Bayesian model selection (BMS) to identify which model is the most likely to have generated the observed data. The process of adjudicating between models essentially balances their goodness of fit against a factor that penalises models for their relative complexity (for review, see Marreiros et al., 2010). BMS yields the evidence for each model—the (posterior) probability of the model given the data—as well as the estimated (posterior) parameter values that reflect the strength of interactions between regions. Relative evidence for all models is used to identify the most likely model, or the best family of models (see Supplementary Material, SM1.5.2). We performed BMS using random effects analysis (Stephan et al., 2009).

### Results

#### Behavioral Results

Analysis of the behavioral data revealed significant effects of both context and noise (Table 1, Figure 2; also Supplementary Material, SM2.1 and Supplementary Figure S1): consistent with its lesser computational burden, participants were more accurate and had faster response times (RTs) for the fast-slow task compared to the same-different one (see Figure 3A, and for effect sizes, see Table 1^[1a,1b]^). Across both contexts, there was also a significant effect of noise: the presence of aperiodic temporal noise in the vibrotactile stimuli decreased accuracy^[1c]^ across both contexts and slowed RT for the same-different context^[1d]^. There was no significant interaction between context and noise.

**Table 1:** Behavioral performance statistics for context (Fast-slow, Same-different), noise (Regular, Noisy), and difference (Different, Same).

Contrast	Factor	Dependent variable	F-statistic	*p*-value	Partial eta square	Text ref.
Noise and context	Context	PC	*F*_(1,15)_ = 87.039	*p* < 0.0001*	0.853	1a
	Noise		*F*_(1,15)_ = 5.352	*p* = 0.0353*	0.263	1c
	Noise * Context		*F*_(1,15)_ = 0.672	*p* = 0.4251	0.043	
(Both contexts)	Context	RT	*F*_(1,15)_ = 28.759	*p* = 0.0001*	0.657	1b
	Noise		*F*_(1,15)_ = 3.154	*p* = 0.0960	0.174	
	Noise * Context		*F*_(1,15)_ = 0.419	*p* = 0.5273	0.027	
Noise and difference	Noise	PC	*F*_(1,15)_ = 0.009	*p* = 0.9256	0.001	1h
	Difference		*F*_(1,15)_ = 4.502	*p* = 0.0509	0.231	1f
	Noise * Difference		*F*_(1,15)_ = 17.927	*p* = 0.0007*	0.544	1g
(Same-different context only)	Noise	RT	*F*_(1,15)_ = 7.240	*p* = 0.0168*	0.326	1d
	Difference		*F*_(1,15)_ = 19.225	*p* = 0.0005*	0.562	1e
	Noise * Difference		*F*_(1,15)_ = 0.286	*p* = 0.6008	0.019	

Proportion correct (PC) was used to assess accuracy and response time (RT) was used to assess speed. *Significant *p*-values.

The lower accuracy in the same-different compared to the faster-slower context could in theory be due to a response bias arising, for example, from a conservative internal standard for the detection of difference. We estimated d-prime (d’), a measure of sensitivity that takes response bias into account (MacMillan and Creelman, 2005). Repeated measures ANOVA re-affirmed significantly lower accuracy for responses in the same-different compared to the fast-slow context (d’ for fast-slow = 1.59, d’ for same-different = 0.72, *F*_(1,15)_ = 36.497, *p* < 0.0001). This suggests that differences in the same-different context were associated with a loss in sensitivity.

Within the same-different task, participants took longer to respond to the same compared to the different trials (Figure 3A)^[1e,f]^ which was associated with a trend-level increase in accuracy (*p* = 0.0509). There was an interesting interaction between noise and difference for accuracy^[1g]^: for same trials, accuracy was greatest when trials were noisy, whereas for different trials accuracy was higher for regular trials (Figure 3B, *p* < 0.0007).

Thus, it appears easier for participants to correctly classify same trials as “same” when they are imbued with temporal noise than when they are pure sinusoids. Conversely, different trials were more likely to be correctly reported when they are regular. These observations can be interpreted by considering the influence of stimulus noise on perceptual accuracy (Figure 3): we return to this issue in the “Discussion” section.

#### Functional Imaging Contrasts

We observed a strong and significant main effect of “context” in our functional imaging data, with several clusters surviving FWE-corrected significance (Table 2 and Supplementary Material, SM2.2.1). All of these effects were in the direction of the same-different over the fast-slow context, again consistent with the additional computational load of this task and mirroring the behavioral results. The strongest effect was expressed in a large cluster in the left inferior frontal gyrus (BA 45; *p* < 0.0001, Supplementary Figure S2A), occupying the mid-ventrolateral PFC (VLPFC). A second effect was observed in the right middle temporal gyrus (BA 21; *p* < 0.0001, Supplementary Figure S2B). Also in accordance with the behavioral results, no significant interaction effects between noise and context were found.

**Table 2:** Significant clusters for the effect of context (fast-slow vs. same-different).

Contrast	Anatomical label	R/L	MNI coordinates			BA	*T*-value	Statistics	
			*X*	*Y*	*Z*			Cluster P_FWE-corr_	Size (voxels)
T: Fast-slow < Same-different	Inferior Frontal Gyrus pars triangularis	L	−36	29	16	45	6.43	<0.0001	41
	Middle Temporal Gyrus	R	51	−25	−14	21	5.59	<0.0001	36

*Significant results of “Faster-slower” < “Same-different” are shown. Standard Montreal Neurological Institute (MNI) coordinates correspond to peak maxima. Size indicates the number of voxels in the cluster. Note that the “Same” trials have been omitted from the same-different contrast as there were no counterpart same trials from the fast-slow contrast*.

We next focussed on effects present within the same-different context (Table 3, Supplementary Material, SM2.2.2). The contrast of different over same trials yielded three distinct clusters, all of which surpassed FWE-corrected significance for both cluster and height statistics. The strongest effect was centered over the left inferior parietal lobule (BA 40; *p* < 0.0001, Figure 4A) and included voxels within the supramarginal and the post-central gyri. Other effects occurred in the PFC, including a strong effect in the left middle frontal gyrus (the dorsolateral PFC, DLPFC, BA 44; *p* < 0.002, Figure 4B). Inspection of the parameter values for these two regions revealed quite distinct responses: whereas the large posterior cluster showed significantly positive values for both different and same trials (with the different greater than same trials, consistent with repetition suppression), the DLPFC cluster only showed non-zero responses to different trials, specific to the “signal trials” (true positives) in this context. A third cluster was located in the midline, centered on the supplementary motor area (BA 6; *p* < 0.008).

**Table 3:** Significant clusters for contrasts within the same-different context.

Contrast	Anatomical label anatomy	R/L	MNI coordinates			BA	*T*-stat	Statistics	
			*X*	*Y*	*Z*			Cluster P_FWE-corr_	Extent
Different > Same	Inferior Parietal Lobule	L	−45	−46	43	40	5.81	0.0001	145
	Middle Frontal Gyrus (DLPFC)	L	−39	14	34	44	5.69	0.002	42
	Supplementary motor area	L	0	23	52	6	4.91	0.008	29
Regular > Noisy	Middle Frontal Gyrus (PFC)	L	−27	44	19	10	4.78	0.016	22
Noise × Difference	Superior Frontal Gyrus	L	−21	23	37	8	4.88	0.010	26

The contrast between regular and noisy trials speaks directly to the representation of perceptual precision. Interestingly, despite the absence of a significant effect of stimulus noise on behavioral accuracy in same-different trials^[1h]^, there existed a strong and specific effect in the imaging data, with a single cluster towards the rostral pole of the left PFC, and in the left DLPFC, for the contrast of regular over noisy trials (BA 10; *p* < 0.016, FWE-corrected, Figure 4C). This cluster lies within a sulcus in rostral PFC (rPFC, BA10), bounded dorsally by the DLPFC. There were no effects approaching significance for the contrast of noisy over regular trials.

The significant interaction between regular-noisy trials and same-different trials present in the behavioral data^[1g]^ motivated analysis of the corresponding interaction in the functional imaging data. We observe a single significant cluster, located within the left superior frontal gyrus (SFG, BA 8, *p* < 0.010 FWE-corrected, Figure 4D, Table 3).

We, therefore, observe four distinct clusters in the left PFC for the main effect of context, the main effect of noise, the main effect of difference and the interaction between noise and difference. Whilst nearby, these four clusters nonetheless reside in distinct sulci. One cluster resides with the VLPFC, and two within the DLPFC.

#### Dynamic Causal Modeling

We next employed DCM to model the interactions between the left inferior parietal lobe (IPL) and the three prefrontal clusters engaged in the second (faster-slower) context (Figure 4E and Supplementary Material, SM3.5). We excluded areas outside of the PFC, such as the supplementary motor area, likely involved in lower level processing and/or preparation for the motor response. All specified dynamic causal models of these data shared a common input base, beginning with stimulus inputs (i.e., vibrotactile stimuli) directed to the left IPL. The effect of regular trials expressed in the rPFC was modeled by an effective connection from IPL to rPFC, modulated by the pure (regular) trials (Figure 4E). Likewise, a connection from the IPL to the DLPFC, modulated by stimulus difference, modeled the effect of difference observed in the DLPFC. Finally, SFG is subjected to the influence of both modulations as the interaction between regular-noisy and same-different trials occurs there. Note that a backward connection was placed here to allow for the diminished response of different compared to same trials to be modeled by the feedback influence of the DLPFC on the IPL.

We specified seven separate models (four bilinear: “Diamond,” “Fork,” “Legs 1,” and “Legs 2”; and three nonlinear: “Stork 1,” “Stork 2,” and “Stork 3”; see Supplementary Material for additional details) on top of this common base that represent serial, parallel or hierarchical processes (see “Materials and Methods” section and Figure 5). As the name suggests, in serial models (both bilinear and nonlinear), information passes in a serial manner from the IPL *via* the rPFC or the DLPFC (or both) *en route* to the SFG. In parallel models, there is a direct effective connection from the IPL to the SFG in parallel to the rPFC and DLPFC connections. Additional modulatory influences are introduced on top of these architectures in order to explain the interaction effect in the SFG. In the nonlinear models (Figure 5, lower row) the modulation of inputs to SFG is mediated by modulation of connections from one area by another (namely DLPFC or rPFC). This activity-dependent modulation can be considered hierarchical. In contrast, in bilinear models (Figure 5, top row), this modulation is attributed directly to experimental inputs (namely, stimulus difference and regularity). In short, both bilinear and nonlinear models allow for context or state-dependent changes in afferents to the SFG: however, nonlinear models consider this state-dependent modulation to be dynamic and activity-dependent. These seven models encompass all possible such serial, parallel and hierarchical arrangements considered separately. Because we sought a parsimonious and non-redundant model space, we did not consider models that combine these basic features (for example both serial and parallel connections).

**Figure 6:**
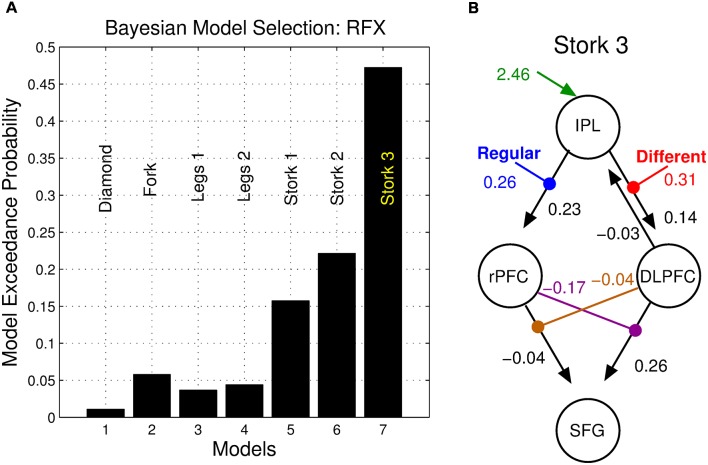
Bayesian model selection (BMS). **(A)** Posterior exceedance probability that any single model is more likely than any other. **(B)** Posterior parameter values of the winning model.

BMS identified the double nonlinear and hierarchical model “Stork 3” as the model with the highest posterior exceedance probability of the seven tested (Figure 6). This model was followed by the other nonlinear models “Stork 2,” and “Stork 1.” The remaining bilinear models embodying serial and parallel motifs performed poorly as they were associated with a considerably lower exceedance probability (Figure 6A).

### Discussion

While being formed, stimulus representations contend with noise in the nervous system, placing an upper bound on the precision of the stimulus representation and confounding any imprecision arising from the properties of the stimulus (Faisal et al., 2008). The precision of the ensuing percept is thus a composite of the stimulus noise and stochastic process in the perceptual system. This is crucial to perceptual inference: not only do we integrate information across modalities by weighting according to relative precision (Jacobs, 1999; Ernst et al., 2000), precision also plays a crucial role in combining new sensory evidence with prior knowledge to inform perceptual beliefs (Friston et al., 1996). However, there must also be a lower bound on precision in many everyday tasks, such that objects that are re-encountered can be recognized as familiar and, conversely, salience can be directed toward novel or surprising parts of the sensorium (Vossel et al., 2014). The modulation of factors influencing perceptual precision is thus context-dependent and under executive control. Using a vibrotactile discrimination task whereby participants made contextual judgments that either implicitly required encoding of a precision estimate (same-different) or not (faster-slower), we identified a constellation of cortical regions predominantly in the left PFC that are engaged in computing, representing and deploying perceptual precision in the service of decision making. By modeling these effects, we observe that effective connectivity amongst these regions is subserved by a hierarchical network whereby activity in left rPFC and DLPFC exert a mutual gating influence on the SFG.

Accuracy is higher and responses are faster for simple magnitude comparisons (fast-slow) than during the detection of difference (same-different). As described by signal detection theory (MacMillan and Creelman, 2005), these two tasks differ in the way stimuli and noise are perceptually represented in “decision space”: although perceptual uncertainty clearly plays a role in all decisions in our experiment (both faster-slower and same-different), the former task can be achieved simply by subtracting the inferred stimulus frequencies. By contrast, in the latter task, perceptual precision is explicitly part of the decision process, so that the perceived magnitude difference is weighed against the precision of each representation (Figures 2, 3). This additional computational burden is reflected in slower reaction times (Figure 3A); the corresponding contextual functional neuroimaging contrast yielded a robust effect in the left IFG pars triangularis (BA 45), which lies within the mid VLPFC and has been implicated in the cognitive control of working memory (Badre and Wagner, 2007), a necessary component of our task. It has also been argued that the mid-VLPFC is involved in the “active retrieval” of information from posterior cortical association areas: active retrieval is required when stimuli in memory “do not bear stable relations to each other and therefore retrieval cannot be automatically driven by strong, stable, and unambiguous stimulus or context relations” (Petrides, 2002). This argument recapitulates the notion that additional neuronal resources are called upon when the ambiguity of perceptual representation becomes an integral aspect of the task at hand and not a mere nuisance factor.

To further understand the neural correlates of perceptual precision, we studied the consequence of degrading the temporal structure of the stimuli, thereby introducing controlled stimulus noise. The contrast of regular > noisy trials in the same-different context showed additional activity in the left rPFC (BA 10, Figure 4), an apex region of the PFC. The rPFC has been associated with a broad variety of executive and integrative functions, including those that pertain to decision making (Koechlin and Hyafil, 2007; Li and Yang, 2012), working memory (Ramnani and Owen, 2004) and context (Simons et al., 2005). The stronger engagement of this region during the regular trials may be indicative of a requirement to account for the relatively high precision of stimulus representations arising from regular vibrations. This might reflect a fundamental role for this region in modifying perceptual stability to optimize the detection of change and surprise (Friston et al., 2012). Greater activity in regular compared to noisy vibrotactile stimuli has been previously observed in other regions of PFC during the explicit detection of stimulus noise (Godde et al., 2010). In our study, detecting the presence of noise was not explicitly required (or reported) but rather an implicit component of task execution. The rPFC may, therefore, encode a generic means of representing perceptual precision rather than a role linked specifically to explicit stimulus decoding. We return to this issue below.

The presence of noisy stimuli in the same-different task was either a help or a hindrance to task performance, depending upon the nature of the trial: consistent with our framing of decision-making in the presence of noise (Figure 2), noise increased the accuracy for same but not different trials. In the case of same trials, stimulus noise may diminish the significance of the slight perception of difference that inevitably arises when encoding stimuli, even when such stimuli are physically identical. The presence of noise thus decreases the chance that such trials are mistakenly classified as different. However, the lower precision also increases the likelihood that the perception of difference associated with truly different trials is rendered subthreshold, increasing their misclassification. This behavioral interaction thus speaks directly to perceptual precision. The corresponding interaction contrast in our functional magnetic resonance imaging (fMRI) data yielded a cluster deep in the sulcus of left DLPFC cortex—the SFG. This finding suggests that in concert with other prefrontal regions such as the rPFC, the SFG may accumulate multiple aspects of decision-relevant evidence and integrate these on the fly.

We employed DCM to model dynamic network computations enacting the interaction of stimulus change and perceptual noise. The key features of the winning model (Stork 3) are nonlinear and hierarchical relationships between the DLPFC, the rPFC and the SFG (Figure 5). The balanced nature of this motif’s structure mirrors the notion that the assessments of precision and stimulus difference mandate a mutual, dynamic exchange during the corresponding same-different task: high values of perceptual precision up-regulate the appreciation of stimulus change and likewise, the perception of change influences the role of precision on decisions. The nonlinear terms that account for the interaction effect ostensibly have an underlying biological basis—a “gating” mechanism, whereby the effective influence of activity from one neural region to another depends on the current activity in a third region. Candidate neural processes capable of underlying this effect include priming of voltage-dependent N-Methyl-D-aspartate (NMDA) channels through partial depolarization by AMPA-mediated synapses, synaptic depression/facilitation or early long-term potentiation (for review, see Stephan et al., 2008). The neural response of the SFG may thus depend on the immediate history of responses of the rPFC (facilitated by regular stimuli) and the DLPFC (facilitated stimulus difference), each influencing the other’s concurrent influence.

The hierarchical organization of networks and information flow has been frequently described across prefrontal regions (Nee and D’Esposito, 2016). The “action-perception cycle” describes the complementary interaction between prefrontal networks of executive memory with a posterior network of perceptual memory, exerting reciprocal influences. This interaction is thought to occur at all levels of the nervous system, engaging neural networks at every hierarchical level of the neocortex (Fuster, 2009). All stages of processing generate internal feedback upon earlier stages, serving to monitor and modulate incoming signals at every stage (Fuster, 2006). Here, we have focused only on the interactions among the constellation of PFC regions identified by the task contrasts. The PFC is thought to constitute the highest level of the cortical hierarchy dedicated to the representation and execution of actions (Fuster, 2001). The analysis of functional and structural hierarchies in PFC is a very active area of research (see Gorbach et al., 2011): to the best of our knowledge, this is the first study of hierarchies of effective connectivity within the human PFC underlying perceptual precision. The predominance of left PFC in this study may be partly due to the fact that all participants in our study were right handed and all stimuli were presented to the right index finger. The lateralization may thus be a consequence of the right-sided stimulus presentation rather than a reflection of hemispheric specialization. Most of our effects were indeed bilateral, although often only exceeding threshold in the left hemisphere (results not shown). Future work could also incorporate premotor regions involved in the task, likely in pre-empting the motor response.

It is important to note that the fast-slow < same-different contrast did not contain the same trials required for the same-different task. Hence, the full stimulus-set used by participants to set their decision-criteria in the same-different context is not present in this contrast. In addition to a substantially lower sensitivity (Supplementary Figure S1), participants possibly adopted a response bias towards responding “same” for the same-different context, reflected in higher accuracy (using proportion correct) for same trials than for different trials. Therefore, the fast-slow < same-different contrast examined in this study, whilst avoiding any confounds due to stimulus differences, is an incomplete comparison of stimulus representation between the two judgments. The neural regions identified from the contrast (IFG pars triangularis and middle temporal gyrus, Supplementary Figure S1) necessarily reflect the perceptual representation of the same-different judgment, and the computational criteria that underlies response bias.

We have framed the performance of our perceptual decision-making task in terms of Bayesian inference, i.e., that decisions depend upon weighting sensory evidence according to perceptual precision (Dayan et al., 1995; Karim et al., 2012). While all percepts accordingly involve both the perceptual value (mean) and the precision, our findings elucidate the manner in which this evidence and its precision are represented and integrated in a hierarchical prefrontal network when required for decision-making. For example, the representation of perceptual precision is associated with greater activity in the rPFC which then gates the effect of other stimulus properties. Our findings build on prior work regarding gain-mediated precision-weighted perceptual inference (Moran et al., 2013) and are consistent with the notion that neuronal activity encodes probability distributions regarding sensory evidence (Dayan et al., 1995; Sanger, 1996; Zemel et al., 1998). However, the application of classic DCM to fMRI data is limited to inferences regarding changes in local mean firing rates. Probabilistic population encoding likely also involves other moments of population activity, such as a direct mapping between the variance of neuronal states and the uncertainty of the perceptual representation (Beck et al., 2008; Shi and Griffiths, 2009). Although there exists a theoretical link between the variance of local population activity and gain control (Marreiros et al., 2010), future work that employs stochastic variants of DCM (Li et al., 2011) could be used to infer higher order moments of neuronal activity (Harrison et al., 2005; Breakspear, 2013) and thus more directly probe the local neural correlates of perceptual precision.

### Ethics Statement

Participants gave written informed consent and the study was approved by the University of New South Wales Human Research Ethics Committee.

## Author Contributions

LG, MK, JH, JM, and MB designed the research and wrote the manuscript. LG. MK, JH, and MB analyzed the data. LG, MK, and MB prepared the figures.

## Conflict of Interest Statement

The authors declare that the research was conducted in the absence of any commercial or financial relationships that could be construed as a potential conflict of interest.

## References

[B2] Bach D. R. Dolan R. J. (2012). Knowing how much you don’t know: a neural organization of uncertainty estimates. Nat. Rev. Neurosci. 13, 572–586. 10.1038/nrn3289 22781958

[B3] Badre D. Wagner A. D. (2007). Left ventrolateral prefrontal cortex and the cognitive control of memory. Neuropsychologia 45, 2883–2901. 10.1016/j.neuropsychologia.2007.06.015 17675110

[B4] Barak O. Tsodyks M. Romo R. (2010). Neuronal population coding of parametric working memory. J. Neurosci. 30, 9424–9430. 10.1523/JNEUROSCI.1875-10.2010 20631171 PMC6632447

[B5] Beck J. M. Ma W. J. Kiani R. Hanks T. Churchland A. K. Roitman J.. (2008). Probabilistic population codes for Bayesian decision making. Neuron 60, 1142–1152. 10.1016/j.neuron.2008.09.021 19109917 PMC2742921

[B6] Behrens T. E. Woolrich M. W. Walton M. E. Rushworth M. F. (2007). Learning the value of information in an uncertain world. Nat. Neurosci. 10, 1214–1221. 10.1038/nn1954 17676057

[B7] Botvinick M. M. (2008). Hierarchical models of behavior and prefrontal function. Trends Cogn. Sci. 12, 201–208. 10.1016/j.tics.2008.02.009 18420448 PMC2957875

[B8] Breakspear M. (2013). Dynamic and stochastic models of neuroimaging data: a comment on Lohmann et al. Neuroimage 75, 270–274. 10.1016/j.neuroimage.2012.02.047 22387473

[B9] Britten K. H. Shadlen M. N. Newsome W. T. Movshon J. A. (1992). The analysis of visual motion: a comparison of neuronal and psychophysical performance. J. Neurosci. 12, 4745–4765. 10.1523/JNEUROSCI.12-12-04745.1992 1464765 PMC6575768

[B10] Daw N. D. Niv Y. Dayan P. (2005). Uncertainty-based competition between prefrontal and dorsolateral striatal systems for behavioral control. Nat. Neurosci. 8, 1704–1711. 10.1038/nn1560 16286932

[B11] Dayan P. Hinton G. E. Neal R. M. Zemel R. S. (1995). The helmholtz machine. Neural Comput. 7, 889–904. 10.1162/neco.1995.7.5.889 7584891

[B12] Eickhoff S. B. Stephan K. E. Mohlberg H. Grefkes C. Fink G. R. Amunts K.. (2005). A new SPM toolbox for combining probabilistic cytoarchitectonic maps and functional imaging data. Neuroimage 25, 1325–1335. 10.1016/j.neuroimage.2004.12.034 15850749

[B13] Engel T. A. Wang X. J. (2011). Same or different? A neural circuit mechanism of similarity-based pattern match decision making. J. Neurosci. 31, 6982–6996. 10.1523/JNEUROSCI.6150-10.2011 21562260 PMC3110065

[B14] Ernst M. O. Banks M. S. (2002). Humans integrate visual and haptic information in a statistically optimal fashion. Nature 415, 429–433. 10.1038/415429a 11807554

[B15] Ernst M. O. Banks M. S. Bülthoff H. H. (2000). Touch can change visual slant perception. Nat. Neurosci. 3, 69–73. 10.1038/71140 10607397

[B16] Fairhall S. L. Ishai A. (2007). Effective connectivity within the distributed cortical network for face perception. Cereb. Cortex 17, 2400–2406. 10.1093/cercor/bhl148 17190969

[B17] Faisal A. A. Selen L. P. Wolpert D. M. (2008). Noise in the nervous system. Nat. Rev. Neurosci. 9, 292–303. 10.1038/nrn2258 18319728 PMC2631351

[B18] Fiorillo C. D. Tobler P. N. Schultz W. (2003). Discrete coding of reward probability and uncertainty by dopamine neurons. Science 299, 1898–1902. 10.1126/science.1077349 12649484

[B19] Friston K. (2005). A theory of cortical responses. Philos. Trans. R. Soc. Lond. B Biol. Sci. 360, 815–836. 10.1098/rstb.2005.1622 15937014 PMC1569488

[B25] Friston K. J. Breakspear M. Deco G. (2012). Perception and self-organized instability. Front. Comput. Neurosci. 6:44. 10.3389/fncom.2012.00044 22783185 PMC3390798

[B24] Friston K. J. Harrison L. Penny W. (2003). Dynamic causal modelling. Neuroimage 19, 1273–1302. 10.1016/S1053-8119(03)00202-7 12948688

[B21] Friston K. J. Holmes A. Poline J.-B. Price C. J. Frith C. D. (1996). Detecting activations in PET and fMRI: levels of inference and power. Neuroimage 4, 223–235. 10.1006/nimg.1996.0074 9345513

[B22] Friston K. J. Holmes A. P. Worsley K. J. Poline J. P. Frith C. D. Frackowiak R. S. (1994a). Statistical parametric maps in functional imaging: a general linear approach. Hum. Brain Mapp. 2, 189–210. 10.1002/hbm.460020402

[B23] Friston K. J. Worsley K. J. Frackowiak R. S. Mazziotta J. C. Evans A. C. (1994b). Assessing the significance of focal activations using their spatial extent. Hum. Brain Mapp. 1, 210–220. 10.1002/hbm.460010306 24578041

[B26] Fuster J. M. (2001). The prefrontal cortex—an update: time is of the essence. Neuron 30, 319–333. 10.1016/s0896-6273(01)00285-9 11394996

[B27] Fuster J. M. (2006). The cognit: a network model of cortical representation. Int. J. Psychophysiol. 60, 125–132. 10.1016/j.ijpsycho.2005.12.015 16626831

[B28] Fuster J. M. (2009). Cortex and memory: emergence of a new paradigm. J. Cogn. Neurosci. 21, 2047–2072. 10.1162/jocn.2009.21280 19485699

[B29] Godde B. Diamond M. E. Braun C. (2010). Feeling for space or for time: task-dependent modulation of the cortical representation of identical vibrotactile stimuli. Neurosci. Lett. 480, 143–147. 10.1016/j.neulet.2010.06.027 20561566

[B30] Gold J. I. Shadlen M. N. (2007). The neural basis of decision making. Annu. Rev. Neurosci. 30, 535–574. 10.1146/annurev.neuro.29.051605.113038 17600525

[B31] Gorbach N. S. Schütte C. Melzer C. Goldau M. Sujazow O. Jitsev J.. (2011). Hierarchical information-based clustering for connectivity-based cortex parcellation. Front. Neuroinform. 5:18. 10.3389/fninf.2011.00018 21977015 PMC3178812

[B32] Grefkes C. Nowak D. A. Eickhoff S. B. Dafotakis M. Küst J. Karbe H.. (2008). Cortical connectivity after subcortical stroke assessed with functional magnetic resonance imaging. Ann. Neurol. 63, 236–246. 10.1002/ana.21228 17896791

[B33] Harris J. A. (2006). “Psychophysical investigations into cortical encoding of vibrotactile stimuli,” in Novartis Foundation Symposium, (New York, NY: John Wiley), 238 https://books.google.com.au/books?hl=en&lr=&id=EcA1avPlxZUC&oi=fnd&pg=PA238&dq=harris+2006+psychophysical+investigations+into+cortical+encoding+&ots=MM_DsMb-2l&sig=ojec0ihhJJaRFGEYEhXH-oNeSMU#v=onepage&q=harris%202006%20psychophysical%20investigations%20into%20cortical%20encoding&f=false 16649718

[B35] Harris J. A. Arabzadeh E. Fairhall A. L. Benito C. Diamond M. E. (2006). Factors affecting frequency discrimination of vibrotactile stimuli: implications for cortical encoding. PLoS One 1:e100. 10.1371/journal.pone.0000100 17183633 PMC1762303

[B34] Harris J. A. Miniussi C. Harris I. M. Diamond M. E. (2002). Transient storage of a tactile memory trace in primary somatosensory cortex. J. Neurosci. 22, 8720–8725. 10.1523/JNEUROSCI.22-19-08720.2002 12351747 PMC6757763

[B36] Harrison L. David O. Friston K. (2005). Stochastic models of neuronal dynamics. Philos. Trans. R. Soc. Lond. B Biol. Sci. 360, 1075–1091. 10.1098/rstb.2005.1648 16087449 PMC1854931

[B37] Heekeren H. R. Marrett S. Ungerleider L. G. (2008). The neural systems that mediate human perceptual decision making. Nat. Rev. Neurosci. 9, 467–479. 10.1038/nrn2374 18464792

[B38] Hegner Y. L. Saur R. Veit R. Butts R. Leiberg S. Grodd W.. (2007). BOLD adaptation in vibrotactile stimulation: neuronal networks involved in frequency discrimination. J. Neurophysiol. 97, 264–271. 10.1152/jn.00617.2006 17065253

[B39] Hernández A. Zainos A. Romo R. (2000). Neuronal correlates of sensory discrimination in the somatosensory cortex. Proc. Natl. Acad. Sci. U S A 97, 6191–6196. 10.1073/pnas.120018597 10811922 PMC18580

[B40] Hsu M. Bhatt M. Adolphs R. Tranel D. Camerer C. F. (2005). Neural systems responding to degrees of uncertainty in human decision-making. Science 310, 1680–1683. 10.1126/science.1115327 16339445

[B41] Huettel S. A. Stowe C. J. Gordon E. M. Warner B. T. Platt M. L. (2006). Neural signatures of economic preferences for risk and ambiguity. Neuron 49, 765–775. 10.1016/j.neuron.2006.01.024 16504951

[B42] Jacobs R. A. (1999). Optimal integration of texture and motion cues to depth. Vision Res. 39, 3621–3629. 10.1016/s0042-6989(99)00088-7 10746132

[B43] Kalberlah C. Villringer A. Pleger B. (2013). Dynamic causal modeling suggests serial processing of tactile vibratory stimuli in the human somatosensory cortex—an fMRI study. Neuroimage 74, 164–171. 10.1016/j.neuroimage.2013.02.018 23435215

[B44] Karim M. Harris J. A. Morley J. W. Breakspear M. (2012). Prior and present evidence: how prior experience interacts with present information in a perceptual decision making task. PLoS One 7:e37580. 10.1371/journal.pone.0037580 22701521 PMC3362626

[B45] Kayser A. S. Buchsbaum B. R. Erickson D. T. D’Esposito M. (2010). The functional anatomy of a perceptual decision in the human brain. J. Neurophysiol. 103, 1179–1194. 10.1152/jn.00364.2009 20032247 PMC2887630

[B46] Kennerley S. W. Walton M. E. Behrens T. E. Buckley M. J. Rushworth M. F. (2006). Optimal decision making and the anterior cingulate cortex. Nat. Neurosci. 9, 940–947. 10.1038/nn1724 16783368

[B47] Knill D. C. Pouget A. (2004). The Bayesian brain: the role of uncertainty in neural coding and computation. Trends Neurosci. 27, 712–719. 10.1016/j.tins.2004.10.007 15541511

[B48] Koechlin E. Hyafil A. (2007). Anterior prefrontal function and the limits of human decision-making. Science 318, 594–598. 10.1126/science.1142995 17962551

[B49] Leff A. P. Schofield T. M. Stephan K. E. Crinion J. T. Friston K. J. Price C. J. (2008). The cortical dynamics of intelligible speech. J. Neurosci. 28, 13209–13215. 10.1523/JNEUROSCI.2903-08.2008 19052212 PMC2613508

[B50] Li B. Daunizeau J. Stephan K. E. Penny W. Hu D. Friston K. (2011). Generalised filtering and stochastic DCM for fMRI. Neuroimage 58, 442–457. 10.1016/j.neuroimage.2011.01.085 21310247

[B51] Li S. Yang F. (2012). Task-dependent uncertainty modulation of perceptual decisions in the human brain. Eur. J. Neurosci. 36, 3732–3739. 10.1111/ejn.12006 23033853

[B52] MacMillan N. A. Creelman C. D. (2005). Detection Theory: A User’s Guide. 2nd Edn. Mahwah, NJ: Psychology Press.

[B53] Marreiros A. C. Stephan K. E. Friston K. J. (2010). Dynamic causal modeling. Scholarpedia 5:9568 10.4249/scholarpedia.9568

[B54] Mechelli A. Price C. J. Noppeney U. Friston K. J. (2003). A dynamic causal modeling study on category effects: bottom-up or top-down mediation? J. Cogn. Neurosci. 15, 925–934. 10.1162/089892903770007317 14628754

[B55] Mesulam M.-M. (1998). From sensation to cognition. Brain 121, 1013–1052. 10.1093/brain/121.6.1013 9648540

[B57] Miller P. Brody C. D. Romo R. Wang X. J. (2003). A recurrent network model of somatosensory parametric working memory in the prefrontal cortex. Cereb. Cortex 13, 1208–1218. 10.1093/cercor/bhg101 14576212 PMC4632206

[B56] Miller E. K. Cohen J. D. (2001). An integrative theory of prefrontal cortex function. Annu. Rev. Neurosci. 24, 167–202. 10.1146/annurev.neuro.24.1.167 11283309

[B58] Moran R. J. Campo P. Symmonds M. Stephan K. E. Dolan R. J. Friston K. J. (2013). Free energy, precision and learning: the role of cholinergic neuromodulation. J. Neurosci. 33, 8227–8236. 10.1523/jneurosci.4255-12.2013 23658161 PMC4235126

[B59] Navajas J. Hindocha C. Foda H. Keramati M. Latham P. E. Bahrami B. (2017). The idiosyncratic nature of confidence. Nat. Hum. Behav. 1, 810–818. 10.1038/s41562-017-0215-1 29152591 PMC5687567

[B60] Nee D. E. D’Esposito M. (2016). The hierarchical organization of the lateral prefrontal cortex. eLife 5:e12112. 10.7554/elife.12112 26999822 PMC4811776

[B61] Noppeney U. Josephs O. Hocking J. Price C. J. Friston K. J. (2008). The effect of prior visual information on recognition of speech and sounds. Cereb. Cortex 18, 598–609. 10.1093/cercor/bhm091 17617658

[B62] Petersen S. E. Sporns O. (2015). Brain networks and cognitive architectures. Neuron 88, 207–219. 10.1016/j.neuron.2015.09.027 26447582 PMC4598639

[B63] Petrides M. (2002). The mid-ventrolateral prefrontal cortex and active mnemonic retrieval. Neurobiol. Learn. Mem. 78, 528–538. 10.1006/nlme.2002.4107 12559832

[B64] Pleger B. Ruff C. C. Blankenburg F. Bestmann S. Wiech K. Stephan K. E.. (2006). Neural coding of tactile decisions in the human prefrontal cortex. J. Neurosci. 26, 12596–12601. 10.1523/jneurosci.4275-06.2006 17135421 PMC2636906

[B65] Pouget A. Beck J. M. Ma W. J. Latham P. E. (2013). Probabilistic brains: knowns and unknowns. Nat. Neurosci. 16, 1170–1178. 10.1038/nn.3495 23955561 PMC4487650

[B66] Preuschhof C. Heekeren H. R. Taskin B. Schubert T. Villringer A. (2006). Neural correlates of vibrotactile working memory in the human brain. J. Neurosci. 26, 13231–13239. 10.1523/jneurosci.2767-06.2006 17182773 PMC6675015

[B67] Ramnani N. Owen A. M. (2004). Anterior prefrontal cortex: insights into function from anatomy and neuroimaging. Nat. Rev. Neurosci. 5, 184–194. 10.1038/nrn1343 14976518

[B68] Romo R. Salinas E. (2003). Flutter discrimination: neural codes, perception, memory and decision making. Nat. Rev. Neurosci. 4, 203–218. 10.1038/nrn1058 12612633

[B69] Rushworth M. F. Behrens T. E. (2008). Choice, uncertainty and value in prefrontal and cingulate cortex. Nat. Neurosci. 11, 389–397. 10.1038/nn2066 18368045

[B70] Sanger T. D. (1996). Probability density estimation for the interpretation of neural population codes. J. Neurophysiol. 76, 2790–2793. 10.1152/jn.1996.76.4.2790 8899646

[B71] Shi L. Griffiths T. L. (2009). “Neural implementation of hierarchical Bayesian inference by importance sampling,” in Advances in Neural Information Processing Systems, eds Bengio Y. Schuurmans D. Lafferty J. Williams C. Culotta A. (Cambridge, MA: MIT Press), 1669–1677.

[B72] Simons J. S. Owen A. M. Fletcher P. C. Burgess P. W. (2005). Anterior prefrontal cortex and the recollection of contextual information. Neuropsychologia 43, 1774–1783. 10.1016/j.neuropsychologia.2005.02.004 16154453

[B73] Smith A. P. Stephan K. E. Rugg M. D. Dolan R. J. (2006). Task and content modulate amygdala-hippocampal connectivity in emotional retrieval. Neuron 49, 631–638. 10.1016/j.neuron.2005.12.025 16476670

[B74] Sörös P. Marmurek J. Tam F. Baker N. Staines W. R. Graham S. J. (2007). Functional MRI of working memory and selective attention in vibrotactile frequency discrimination. BMC Neurosci. 8:48. 10.1186/1471-2202-8-48 17610721 PMC1925104

[B76] Stephan K. E. Kasper L. Harrison L. M. Daunizeau J. den Ouden H. E. Breakspear M.. (2008). Nonlinear dynamic causal models for fMRI. Neuroimage 42, 649–662. 10.1016/j.neuroimage.2008.04.262 18565765 PMC2636907

[B75] Stephan K. E. Marshall J. C. Penny W. D. Friston K. J. Fink G. R. (2007). Interhemispheric integration of visual processing during task-driven lateralization. J. Neurosci. 27, 3512–3522. 10.1523/jneurosci.4766-06.2007 17392467 PMC2636903

[B77] Stephan K. E. Penny W. D. Daunizeau J. Moran R. J. Friston K. J. (2009). Bayesian model selection for group studies. Neuroimage 46, 1004–1017. 10.1016/j.neuroimage.2009.03.025 19306932 PMC2703732

[B78] Stephan K. E. Penny W. D. Moran R. J. den Ouden H. E. Daunizeau J. Friston K. J. (2010). Ten simple rules for dynamic causal modeling. Neuroimage 49, 3099–3109. 10.1016/j.neuroimage.2009.11.015 19914382 PMC2825373

[B80] Summerfield C. Egner T. Greene M. Koechlin E. Mangels J. Hirsch J. (2006). Predictive codes for forthcoming perception in the frontal cortex. Science 314, 1311–1314. 10.1126/science.1132028 17124325

[B79] Summerfield C. Koechlin E. (2008). A neural representation of prior information during perceptual inference. Neuron 59, 336–347. 10.1016/j.neuron.2008.05.021 18667160

[B81] Tobler P. N. O’Doherty J. P. Dolan R. J. Schultz W. (2007). Reward value coding distinct from risk attitude-related uncertainty coding in human reward systems. J. Neurophysiol. 97, 1621–1632. 10.1152/jn.00745.2006 17122317 PMC2637604

[B82] Vossel S. Mathys C. Daunizeau J. Bauer M. Driver J. Friston K. J.. (2014). Spatial attention, precision, and bayesian inference: a study of saccadic response speed. Cereb. Cortex 24, 1436–1450. 10.1093/cercor/bhs418 23322402 PMC4014178

[B83] Wang X.-J. (2008). Decision making in recurrent neuronal circuits. Neuron 60, 215–234. 10.1016/j.neuron.2008.09.034 18957215 PMC2710297

[B84] Wang X.-J. (2012). Neural dynamics and circuit mechanisms of decision-making. Curr. Opin. Neurobiol. 22, 1039–1046. 10.1016/j.conb.2012.08.006 23026743 PMC4065788

[B1] Yu A. J. Dayan P. (2005). Uncertainty, neuromodulation, and attention. Neuron 46, 681–692. 10.1016/j.neuron.2005.04.026 15944135

[B85] Zemel R. S. Dayan P. Pouget A. (1998). Probabilistic interpretation of population codes. Neural Comput. 10, 403–430. 10.1162/089976698300017818 9472488

[B86] Zwislocki J. J. Relkin E. M. (2001). On a psychophysical transformed-rule up and down method converging on a 75% level of correct responses. Proc. Natl. Acad. Sci. U S A 98, 4811–4814. 10.1073/pnas.081082598 11287663 PMC31916

