# Supplementary material for: Hierarchical and Nonlinear Dynamics in Prefrontal Cortex Regulate the Precision of Perceptual Beliefs
Source: Front Neural Circuits. 2019 Apr 24;13:27. doi: 10.3389/fncir.2019.00027 (PMC6491505; doi:10.3389/fncir.2019.00027)
Supplement: Supplementary file 1 [file Data_Sheet_1.pdf]

Supplementary Material

SM1. Materials and Methods

SM1.1 Equipment

Mechanical vibrotactile stimuli were delivered with a vibrotactile stimulator (Dancer Design, St. Helens, UK), which used piezoelectric bender elements. Mechanical contact to the skin was made via a flat plastic tip 8 mm in diameter which was mechanically coupled to the bender element. A static surround with a hole of 10 mm in diameter limited the stimulation to a region just under the contactor. All stimuli took the form of sinusoidal displacement waveforms. The vibrotactile discrimination task was written in Matlab (version 2007b, Mathworks), using the Psychophysics Toolbox extensions (Brainard 1997, Kleiner, Brainard et al. 2007) and used a National Instruments card (USB-6259, National Instruments) to drive the equipment. The stimulators had a dynamic range from 1 to 500 Hz with amplitudes up to 1 mm peak-to-peak (below 200 Hz) when supplied with a 10 V peak-to-peak input signal.

SM1.2 Stimuli and trial structure

Tactile stimuli were 512 ms in duration with approximate amplitude of 280 µm peak-to-peak. On each given trial, the participants compared two consecutive vibrations, separated by an interstimulus interval (ISI) of 600 ms. One of the vibrations was a set base frequency (32, 34 or 36 Hz), the other a comparison frequency. The participant’s right index finger pad was placed on the vibrotactile probe. The participant’s left middle and index fingers were placed on a keyboard’s left and right arrow response keys, respectively. Participants were prompted to answer each context question in a Yes/No fashion. The left/right position of Yes and No was counterbalanced across participants. Participants had two seconds to respond from the onset of the second vibration (Stim2) and were instructed to answer as quickly and accurately as possible.

SM1.3 Main behavioural task

Participants completed a partial 2 (context) × 2 (noise) × 3 (difficulty) factorially designed vibrotactile task. This task was first performed as a purely behavioural study directly after the titration: The reported behavioural data derive from this experiment. Within one week, it was then performed during acquisition of functional neuroimaging data.

Each task trial contained a base frequency of 32, 34 or 36 Hz, and a comparison frequency equal to the base frequency plus or minus a value determined by the participant's performance during the titration procedure (easy, medium and hard frequency difference values). The selection of base frequency, the value of the comparison frequency, and the presentation order of base and comparison vibrations were pseudorandomly presented. To create the stimulus noise factor, the temporal structure of the two vibrations was degraded by adding independent Gaussian-distributed values (mean = 0) to the wavelength of each cycle of the sine wave (Harris, Arabzadeh et al. 2006). We added 8% noise, meaning that the standard deviation of the cycle length within the vibration equalled 0.08 of the base cycle length. For example, a 40 Hz vibration was comprised of cycles with mean length 25 ms and standard deviation of 2 ms.

The contextual (task) factor was created by asking participants to perform either a fast-slow or a same-different comparison. For fast-slow context sessions, participants were informed that there was always a faster vibration. They were instructed to answer the question "Is the 2nd vibration faster?" as a Yes/No response. For this context, there were five trial-types; easy, medium, hard, easy-noisy, medium-noisy. Pilot testing showed that participants performed no more accurate than chance with the addition of noise to the hard trials. For the same-different context sessions, participants were informed that half of the presented vibration pairs were the Same, and the other half were Different. They were instructed to answer the question "Are the vibrations different?" as a Yes/No response. For the same-different context, there were six trial-types; easy-different, medium-different, easy-noisy-different, easy-same, medium-same, easy-noisy-same. Here, we did not add noise to the medium trials in the second context because pilot behavioural data showed that subjects performed with no greater than chance levels of accuracy. Table S1 shows the factorial structure of the task.

Both easy-same and medium-same trials included two identical stimuli: In half of these trials, the base frequency was repeated. Otherwise the easy/medium comparison frequency was presented.

Easy-noisy-same trials contained two identical noise-embedded stimuli to compliment the easy-noisy-different trials. Imaging data were acquired across four separate blocks, each separated by a brief rest. The presentation order for each session was alternated and counterbalanced across participants. There were 24 trials for each trial-type across the four blocks. The trial-types facilitated a number of contrasts that revealed significant neural regions to inform the DCM modelling. There were Fast-slow/Same-different, Regular/Noisy and Different/Same.

Trial-type	Trial-type name	Stimuli feature of vibration pair trial-types			
		**Task difficulty**	**Noise**	**Context**	**Difference**
EC1	Easy	Easy	Regular	Fast-slow	Different
MC1	Medium	Medium	Regular	Fast-slow	Different
HC1	Hard	Hard	Regular	Fast-slow	Different
ENC1	Easy-noisy	Easy	Noisy	Fast-slow	Different
MNC1	Medium-noisy	Medium	Noisy	Fast-slow	Different
EC2	Easy-different	Easy	Regular	Same-different	Different
MC2	Medium-different	Medium	Regular	Same-different	Different
ENC2	Easy-noisy-different	Easy	Noisy	Same-different	Different
SEC2	Easy-same	Easy	Regular	Same-different	Same
SMC2	Medium-same	Medium	Regular	Same-different	Same
SNC2	Easy-noisy-same	Easy	Noisy	Same-different	Same

Table S1. Abbreviations of trial-types used in reported behavioural and fMRI contrasts. Combinations of trial-types used in the repeated measures ANOVAs for the behavioural results and fMRI contrasts. Additional trial-types hard ("HC1") and medium-noisy ("MNC1") were not examined in this study, and not listed in the table.

SM1.4 MRI acquisition and preprocessing

Participants were scanned using a Philips (Achieva X) 3.0-Tesla scanner (Philips Medical Systems, Best, The Netherlands). Functional images were acquired using T2*-weighted gradient echo-planar sequences (29 axial slices, repetition time/echo time: 2000/30 ms, 90° flip angle, matrix size: 112 × 128, field of view: 240 mm, voxel size: 2.14 × 2.73 × 4.5 mm, no gap). A T1-weighted structural images were acquired coronally (repetition time/echo time: 6.39/2.9 ms, 8° flip angle, matrix size: 256 × 256, field of view: 256 × 256 × 180 mm, voxel size: 1 × 1 × 1 mm, no gap). A liquid crystal display projector back-projected the visual stimuli of context instructions, fixation point and response screen onto a screen positioned at the end of the scanner bed. Stimuli were delivered via the vibrotactile device to the right index finger. The participant's index and

middle fingers were positioned on the left and right buttons of a cedrus lumina box in which to make responses. The task was identical to that used for the behavioural analysis except for the inter-trial intervals which were jittered pseudorandomly between 6 and 12 seconds to temporally decorrelate the evoked haemodynamic responses between trials. As with the behavioural version, the fMRI main task was conducted over four separate sessions separated by a short break. To shorten the duration of each session, the distinctly slow and fast vibrations were not included.

Preprocessing of dynamic images included realignment of scans to the first of each session and normalisation into standard Montreal Neurological Institute (MNI) space, re-sampling of the image into 3 × 3 × 3 mm isotropic voxels, and smoothing with an 8 × 8 × 8 mm Gaussian kernel using statistical parametric mapping SPM8 software (Wellcome Department of Imaging Neuroscience, University College London, London, UK, http://www.fil.ion.ucl.ac.uk/spm/). Statistical analysis of the time series of images was conducted using the General Linear Model with regressors modelling each of the factor components as a 200 ms boxcar function starting 200 ms before the participant's response. These were convolved with the canonical haemodynamic response function. The model also included realignment parameters and regressors for session specific offsets and drift. For each participant, statistical parametric maps of the T statistic were generated from linear contrasts for each individual event listed in Table 1. Group-level, random-effects analyses were performed by entering these individual subject contrast images into a flexible factorial ANOVA including a subject factor and non-sphericity correction for repeated measures. Although our experiment has a 2 (context) × 2 (noise) × 3 (difficulty) factorial design, for reasons of simplicity we do not presently study the task difficulty factor (see Table S1 for behavioural statistics on task difficulty), but rather focus on context and noise (and their interaction). In the second (same-different) task there also exists an additional stimulus factor, namely Different versus Same trials: We hence also investigate this factor within this context.

Unless stated otherwise, we use an uncorrected height threshold of $p<0.00005$ and a spatial extent of 20 voxels and report clusters that survive a family-wise error (FWE) $p<0.05$ (Friston, Worsley et al. 1993). We use corrected cluster-level statistics for all imaging results as these are more sensitive than voxel-wise height thresholds, whilst still ensuring family-wise error control for whole brain analyses when using a conservative threshold (Friston, Worden et al. 1993, Friston,

Holmes et al. 1996). Cluster locations were identified using the SPM Anatomy toolbox (Eickhoff, Stephan et al. 2005).

SM.1.5 Dynamic Causal Modelling

SM1.5.1. Time series extraction

For each participant, BOLD signal time courses were extracted from each ROI using a sphere of 6 mm radius centred at the voxel showing the group-wise maximum contrast. Given the voxel resolution was 2×2×2 mm, each ROI comprised approximately 120 voxels. The principle component (essentially the mean) over all voxels in each sphere served as the ROI summary time series. An iterative maximum likelihood algorithm was used to maximise the likelihood of the model (the fitted time BOLD series) given these subject-specific data (Stephan, Marshall et al. 2007).

SM1.5.2. Bayes model selection (BMS)

Model estimation and comparison was performed using the variational Bayes algorithm in DCM12, implemented in SPM12b. For a given model, DCM uses the neural state equation to generate predicted BOLD signals via the integration of the neural dynamics and the well-established hemodynamic model (Friston, Harrison et al. 2003). By optimising the fit of the predicted BOLD signals to the observed data, DCM estimates the model parameters and their posterior distribution via Bayesian model inversion.

For model comparison at the group level, we applied Bayesian model selection (BMS) with a random-effect analysis to select the optimal model (Stephan, Penny et al. 2009). The BMS is based on the Free energy principle, which is an approximation of the model evidence representing the likelihood of a model given the data. The free energy principle penalises the likelihood of the model by the model complexity (Penny, Stephan et al. 2004, Penny 2012). A penalty for complexity is required, because as complexity increases, model fit increases monotonically (Stephan, Kasper et al. 2008). In DCM, model complexity is estimated as the divergence between the prior and posterior model parameters, also known as "surprise" (Friston, Mattout et al. 2007). The quotient (likelihood to surprise) yields a single scalar value called the "free energy" (Friston

2010). After BMS, an expected probability score is produced for each model. The higher the expected probability of a model, the greater the likelihood that particular model parsimoniously accounts for the observed data. An exceedance probability estimate is also derived, representing the likelihood that any single model is more likely than all others.

SM2. Results

SM2.1 Behavioural results

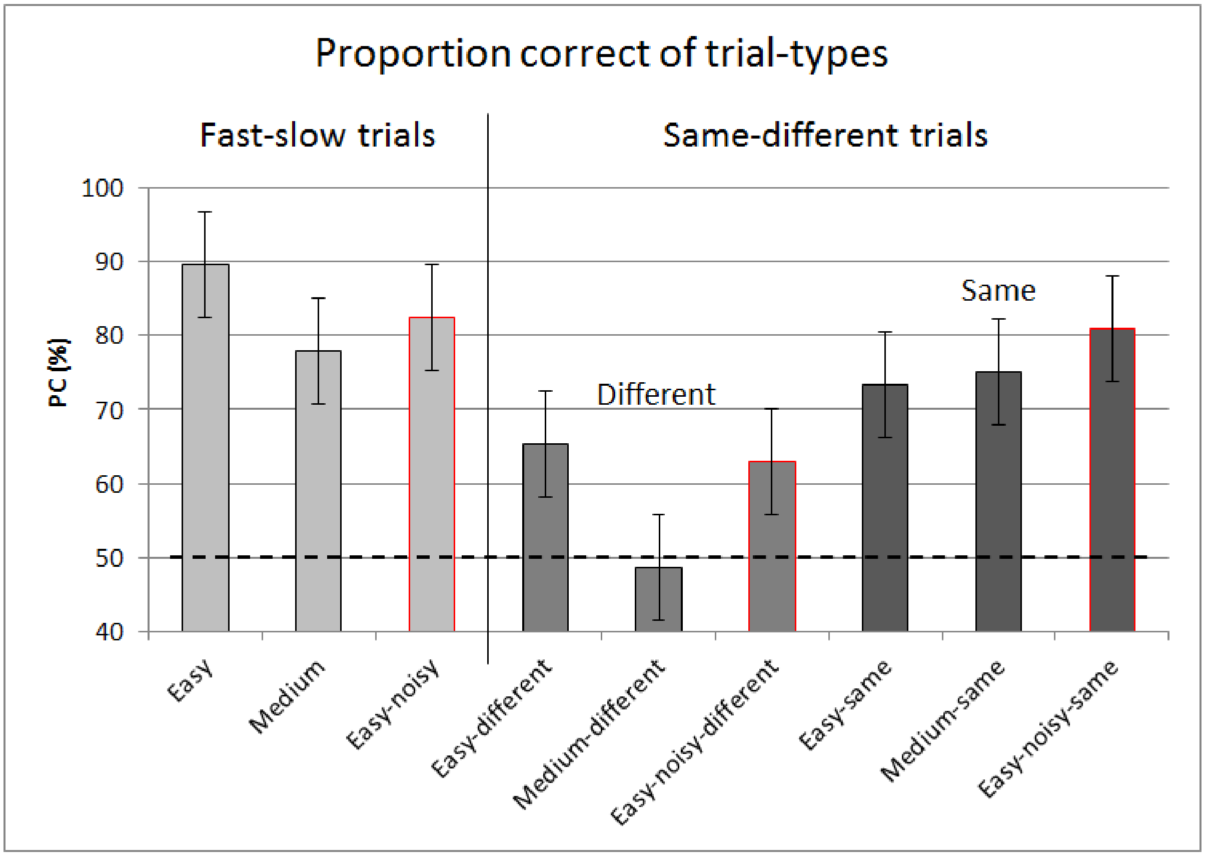

Figure S1. Proportion correct (PC) for reported trial-types comprised of different task difficulties, noise and stimulus difference. Trials to the left of the vertical line are from the fast-slow context trials. Trials to the right are from the same-different context trials. Trial-types of paired stimuli with embedded aperiodic temporal noise are shown with bars boxed in red. Vertical bars represent within-subject standard error of the mean. The dashed horizontal line indicates chance performance. Figure 2 of the main text shows the accuracy for the easy (regular and noisy; same and different) trials in the faster-slower context. Error bars represent within-subject standard error of the mean.

SM2.2 Functional imaging results

SM2.2.1. Main effect of context

We observed a strong and significant main effect of “context” in our functional imaging data, with two large clusters surviving FWE-corrected significance at both the peak and cluster-level (Table 2). This contrast was constructed by pooling over all conditions present in both contexts (easy different, medium different, easy-noisy different). The strongest effect was expressed in the left inferior frontal gyrus (BA 45; p<0.0001, Figure S2A), occupying the mid-ventrolateral prefrontal cortex (VLPFC). A second effect was observed in the right middle temporal gyrus (BA 21; p<0.0001, Figure S2B).

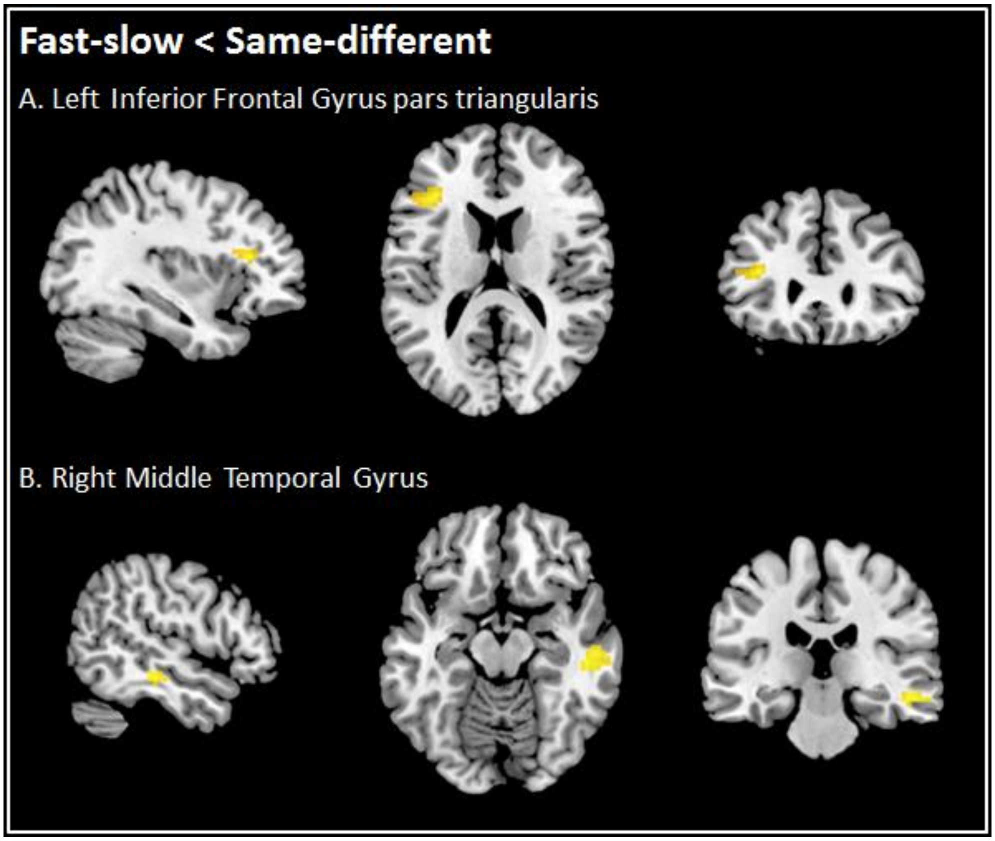

Figure S2. Significant clusters for the effect of context (fast-slow versus same-different). Significant results of "Faster-slower" < "Same-different" are shown. (A) Left Inferior frontal gyrus G; (B) Right Middle temporal gyrus. All clusters p<0.05, FWE corrected.

SM2.2.2. Contrasts within same-different context.

Contrasts were then performed within the second context. For the different>same trial contrast, the strongest effect was centred over the left inferior parietal lobule (BA 40; $p<0.0001$, FWE corrected) and included voxels within the supramarginal and the post-central gyri. Other effects associated with this contrast occured in the left prefrontal cortex, including a strong effect in the left middle frontal gyrus (the dorsolateral prefrontal cortex, DLPFC, BA 44; $p<0.002$, FWE corrected) and left supplementary motor area ($p<0.008$, FWE corrected).

The contrast between regular and noisy trials was constructed from the trials shown in Figure 2 of the main text (easy - regular and noisy; same and different). There existed a single cluster towards the rostral pole of the left PFC, in the left middle frontal gyrus (BA 10; $p<0.016$, FWE-corrected). This cluster lies within a sulcus in rostral prefrontal cortex (rPFC, BA10), bounded dorsally by the DLPFC. There were no effects approaching significance for the contrast of noisy over regular trials.

The interaction between regular-noisy trials and same-different trials yielded a single significant cluster, located within the left superior frontal gyrus (BA 8, $p<0.010$ FWE-corrected).

Note that this same cluster was also significant whether using all available (noise and noise-free) trials within the second context ($p<0.010$ FWE-corrected, 26 voxels) or only using the easy trials $p<0.039$ FWE-corrected, 53 voxels).

SM3.5 Dynamical causal modelling: Model construction from GLM results

SM3.5.1 Choice of regions of interest

We restricted the DCM analysis exclusively to explain the effects observed in the same-different task. The different>same contrast identified the left IPL as an early sensory region in the task . That is, although different trials elicited a stronger effect than same trials, the parameter estimates for the latter were nonetheless clearly non-zero. This is consistent with repetition-suppression in sensory cortex. For our models, this region hence always receives the direct trial inputs. The remaining regions in our network are the three prefrontal regions that show significant effects for contrasts within the same-different context, namely the effects of difference in the DLPFC, of stimulus regularity in the rPFC, and their interaction in the SFG. The regions receive (direct or indirect) inputs from IPL via intrinsic connections, the nature of which differs between models.

The experimental conditions that modulate the strength of these connections are (1) Whether the vibration stimulus pairs were the same or different, and (2) The absence or presence of aperiodic temporal noise in the stimuli. These experimental conditions enter the model as modulating inputs and are permitted to modulate connection strengths either directly (through bilinear terms) (Stephan, Penny et al. 2010) or indirectly, via nonlinear effects mediated by other regions (Stephan, Kasper et al. 2008).

SM3.5.2 Model space specification

Neuronal activity of each ROI is represented in DCM by a single (hidden) state variable, perturbed by the experimentally controlled inputs (Stephan, Kasper et al. 2008). We constrained our specifications to those specific models that could explain the causes of our fMRI data (Stephan, Penny et al. 2010). The models were constrained by the following principles: (1) Driving inputs – the trials – elicited direct influences via the IPL; (2) Intrinsic connections existed between the IPL and the three prefrontal regions either directly or via one of the other regions, and (3) Context-dependent modulators were introduced in a manner that could parsimoniously explain the effects of the data. No superfluous influences were introduced. For example, the connection between IPL and rPFC was modulated by stimulus regularity – to allow that contrast to be modelled – but not stimulus difference, which did not exert an effect there.

The contrast of different>same trials revealed activity in the IPL, the DLPFC, and the supplementary motor area. However, since we are interested in the higher-level processing of vibrotactile stimuli, the supplementary motor area was excluded from further analysis. The average parameter estimates (indicative of neural activity) for the different trials was greater than that of the same trials (results not shown). Hence, all models include a forward connection from the IPL (lowest-level of the model hierarchy) to the higher cortical region of the DLPFC. To account for the greater activity in the IPL for this contrast, all models also include a backward connection from the DLPFC to the IPL. That is, in the presence of different trials the DLPFC is permitted by this model feature to feedback increased activity to the IPL. Following the same principle, all models also contained a forward connection from the IPL to the rPFC, the latter region identified from the contrast of regular>noisy trials. The modulation of this connection allows the presence of regular stimuli, as opposed to aperiodic noise, to increase the activity of the rPFC.

All models thus share a common base consisting of these three regions and their modulated connections. The fourth region, the SFG, sits at the top of the hierarchy for each model. The SFG expressed the noise by stimulus difference interaction. The seven models differ in the manner in which the main effects interact through model motifs to explain this interactions effect.

The connections between each of the four regions differ across the seven models generated (Figure 4), and embody whether the system can be considered a serial, parallel, or hierarchical motif:

- The first model ("Diamond") has a connection from the DLPFC to the SFG and a connection from the rPFC to the SFG. This model embodies parallel processing: Stimulus-specific differences in each of DLPFC and rPFC are combined and passively inherited in SFG to yield the interaction effect.
- The second model ("Fork") has a direct connection from the IPL to the SFG, independently modulated by the factors of difference and regularity. This model hence embodies a completely parallel mode of computation, with no interactions within PFC, but rather the hierarchy merely imposed by the relative number of modulating factors, each of which is enacted by unspecified regions.

- The third and fourth ("Legs 1" and "Legs 2" are hierarchical bilinear models whereby the main effect in one or both of the DLPFC and the rPFC are modulated by the opposing effect while connecting to the SFG. The modulating effect is *not* specified within the motif, but rather imposed as an external term. "Legs 1" has a connection from the DLPFC to the SFG, modulated by the presence of regularity. Conversely, "Legs 2" has a connection from the rPFC to the SFG modulated by the presence of trial difference.
- The fifth and sixth models ("Stork 1" and "Stork 2") are self-contained, hierarchical models in the sense that the contextual effects in one region (rPFC or DLPFC) gates, via a nonlinear term, effective connections from other to the SFG. Note that whereas the first two models are classic "bilinear" DCM, this model employs nonlinear DCM.
- The seventh model "Stork 3" is a hierarchical and nonlinear model, which is a symmetric combination of Storks 1 and 2 such that rPFC and DLPFC operate at the same level of the hierarchy.

These seven models hence differ across four different classes – serial, parallel, bilinear hierarchical, nonlinear hierarchical and double-nonlinear hierarchical, and they all embody minimal possible causes of our data (Figure 4), such that rPFC is sensitive to regularity, IPL and DLPFC are sensitive to difference, and SFG is sensitive to both difference and regularity.

REFERENCES

Brainard, D. H. (1997). "The Psychophysics Toolbox." Spat Vis **10**(4): 433-436.
Eickhoff, S. B., K. E. Stephan, H. Mohlberg, C. Grefkes, G. R. Fink, K. Amunts and K. Zilles (2005). "A new SPM toolbox for combining probabilistic cytoarchitectonic maps and functional imaging data." Neuroimage **25**(4): 1325-1335.
Friston, K. (2010). "The free-energy principle: a unified brain theory?" Nature Reviews Neuroscience **11**(2): 127.
Friston, K. J., L. Harrison and W. Penny (2003). "Dynamic causal modelling." Neuroimage **19**(4): 1273-1302.
Friston, K. J., A. Holmes, J. B. Poline, C. J. Price and C. D. Frith (1996). "Detecting activations in PET and fMRI: levels of inference and power." Neuroimage **4**(3 Pt 1): 223-235.
Friston, K. J., J. Mattout, N. Trujillo-Barreto, J. Ashburner and W. Penny (2007). "Variational free energy and the Laplace approximation." Neuroimage **34**(1): 220-234.
Friston, K. J., J. Worden, R. Frackowiak, J. C. Mazziotta and A. C. Evans (1993). "Assessing the significance of focal activations using their spatial extent." Human Brain Mapping **1**(3): 210-220.

Friston, K. J., K. J. Worsley, R. S. J. Frankowiak, J. C. Mazziotta and A. C. Evans (1993). "Assessing the significance of focal activations using their spatial extent." Human Brain Mapping **1**(3): 210-220.

Harris, J. A., E. Arabzadeh, A. L. Fairhall, C. Benito and M. E. Diamond (2006). "Factors affecting frequency discrimination of vibrotactile stimuli: implications for cortical encoding." PLoS ONE **1**: e100.

Kleiner, M., D. Brainard and D. Pelli (2007). ""What's new in Psychtoolbox-3?"." Perception **36: ECVP Abstract Supplement**.

Penny, W. D. (2012). "Comparing dynamic causal models using AIC, BIC and free energy." Neuroimage **59**(1): 319-330.

Penny, W. D., K. E. Stephan, A. Mechelli and K. J. Friston (2004). "Comparing dynamic causal models." Neuroimage **22**(3): 1157-1172.

Stephan, K. E., L. Kasper, L. M. Harrison, J. Daunizeau, H. E. den Ouden, M. Breakspear and K. J. Friston (2008). "Nonlinear dynamic causal models for fMRI." Neuroimage **42**(2): 649-662.

Stephan, K. E., J. C. Marshall, W. D. Penny, K. J. Friston and G. R. Fink (2007). "Interhemispheric integration of visual processing during task-driven lateralization." J Neurosci **27**(13): 3512-3522.

Stephan, K. E., W. D. Penny, J. Daunizeau, R. J. Moran and K. J. Friston (2009). "Bayesian model selection for group studies." Neuroimage **46**(4): 1004-1017.

Stephan, K. E., W. D. Penny, R. J. Moran, H. E. den Ouden, J. Daunizeau and K. J. Friston (2010). "Ten simple rules for dynamic causal modeling." Neuroimage **49**(4): 3099-3109.
